# The impact of diabetes, education and income on mortality and cardiovascular events in hypertensive patients: A cohort study from the Swedish Primary Care Cardiovascular Database (SPCCD)

**DOI:** 10.1371/journal.pone.0237107

**Published:** 2020-08-03

**Authors:** Tobias Andersson, Miriam Pikkemaat, Linus Schiöler, Per Hjerpe, Axel C. Carlsson, Per Wändell, Karin Manhem, Thomas Kahan, Jan Hasselström, Kristina Bengtsson Boström

**Affiliations:** 1 School of Public Health and Community Medicine, Institute of Medicine, Sahlgrenska Academy, University of Gothenburg, Göteborg, Sweden; 2 Närhälsan Norrmalm Health Centre, Skövde, Sweden; 3 Department of Clinical Sciences, Lund University, Malmö, Sweden; 4 R&D Centre Skaraborg Primary Care, Skövde, Sweden; 5 Division of Family Medicine and Primary Care, Department of Neurobiology, Care Sciences and Society, Karolinska Institutet, Stockholm, Sweden; 6 Department of Molecular and Clinical Medicine, Institute of Medicine, Sahlgrenska Academy, University of Gothenburg, Göteborg, Sweden; 7 Division of Cardiovascular Medicine, Department of Clinical Sciences, Danderyd Hospital, Karolinska Institutet, Stockholm, Sweden; 8 Academic Primary Care Center, Region Stockholm, Stockholm, Sweden; University of Bologna, ITALY

## Abstract

**Objective:**

In this study we aimed to estimate the effect of diabetes, educational level and income on the risk of mortality and cardiovascular events in primary care patients with hypertension.

**Methods:**

We followed 62,557 individuals with hypertension diagnosed 2001–2008, in the Swedish Primary Care Cardiovascular Database. Study outcomes were death, myocardial infarction, and ischemic stroke, assessed using national registers until 2012. Cox regression models were used to estimate adjusted hazard ratios of outcomes according to diabetes status, educational level, and income.

**Results:**

During follow-up, 13,231 individuals died, 9981 were diagnosed with diabetes, 4431 with myocardial infarction, and 4433 with ischemic stroke. Hazard ratios (95% confidence intervals) for diabetes versus no diabetes: mortality 1.57 (1.50–1.65), myocardial infarction 1.24 (1.14–1.34), and ischemic stroke 1.17 (1.07–1.27). Hazard ratios for diabetes and ≤9 years of school versus no diabetes and >12 years of school: mortality 1.56 (1.41–1.73), myocardial infarction 1.36 (1.17–1.59), and ischemic stroke 1.27 (1.08–1.50). Hazard ratios for diabetes and income in the lowest fifth group versus no diabetes and income in the highest fifth group: mortality 3.82 (3.36–4.34), myocardial infarction 2.00 (1.66–2.42), and ischemic stroke 1.91 (1.58–2.31).

**Conclusions:**

Diabetes combined with low income was associated with substantial excess risk of mortality, myocardial infarction and ischemic stroke among primary care patients with hypertension.

## Introduction

Hypertension and diabetes are common conditions and are important risk factors both separately and in combination for later development of cardiovascular disease and premature death [1, 2]. Worldwide, hypertension is affecting more than a billion people [3] and the prevalence in Sweden has been estimated to approximately 30% [4]. In 2017, almost half a billion were estimated to be living with diabetes globally [5], and from an international viewpoint the Swedish prevalence of 5% is relatively low [6]. Coexisting type 2 diabetes and hypertension is common and both diseases are usually treated in primary health care.

Furthermore, the connection between type 2 diabetes and heart failure [7], and increased risk of cardiovascular disease [8] is well established. Increased risk of cardiovascular disease has also been shown among patients with prediabetes, where adverse outcomes have been associated with endothelial dysfunction and increased inflammatory tone [9, 10]. Regarding the risk of stroke, type 2 diabetes has been observed to confer an increased risk of subclinical episodes of atrial fibrillation and stroke also among young patients with low thrombo-embolic risk [11].

Treatment of hypertension and other risk factors aim to lower the risk of cardiovascular complications and premature death in patients with type 2 diabetes, and large benefits are seen when multiple risk factors are addressed simultaneously [12]. This has recently been shown in a Swedish nationwide registry based study where people with type 2 diabetes and in-range control of 5 risk factors had little or no excess risk of death, myocardial infarction or stroke [13]. In addition to classic established risk factors, studies in the general population have shown an association between low socioeconomic status and the risk of cardiovascular disease and death in people without [14, 15] or with diabetes [16, 17], also in a high income country with subsidized universal healthcare [18].

The interplay between diabetes and socioeconomic status in relation to cardiovascular risk among individuals with hypertension has only been studied to a limited extent [19], and not in a primary care setting. There is also a gap of knowledge concerning the relative importance of diabetes and socioeconomic status as risk factors for mortality and adverse cardiovascular outcomes in that context. Therefore, the aim of this study was to estimate the effect of diabetes, educational level and income on the risk of mortality, myocardial infarction and ischemic stroke among patients in primary care with hypertension.

## Materials and methods

### The Swedish Primary Care Cardiovascular Database and study setting

The Swedish Primary Care Cardiovascular Database (SPCCD) has been described in detail previously [20] and includes 74,751 individuals aged 30 years or older with registered diagnosis of hypertension (International Classification of Disease (ICD) 10 codes I10, I13P and I15) in primary care 2001–2008. The SPCCD includes data from 48 primary health care centers located in the urban area of south-western Stockholm and the rural area of Skaraborg. These areas include approximately 600,000 inhabitants. The Swedish personal identification number [21] was used to link the individuals’ computerized medical records from primary care with population-based national registers. Data extracted from the medical records included body weight and length, body mass index (BMI), systolic blood pressure (SBP), diastolic blood pressure (DBP), smoking habits, fasting total cholesterol, low density lipoprotein (LDL), high density lipoprotein (HDL), triglycerides, creatinine, prescribed drugs, as well as diagnoses in primary care: atrial fibrillation/flutter (AF, ICD 10: I48), congestive heart failure (HF, I50), diabetes mellitus (E10–11, E14), ischemic heart disease (IHD, I20–25), cerebrovascular disease (CVD, I60–69) and transient ischemic cerebral attack (TIA, G45). Data from the National Patient Register [22] included ICD-10 codes of hospital in- and outpatient care 1996–2012 regarding AF, HF, diabetes mellitus, IHD, CVD, TIA, myocardial infarction (I21), ischemic stroke (I63), kidney failure (N18), cancer (C00–97), and procedure codes regarding percutaneous coronary intervention (PCI, code FNG) and coronary artery bypass grafting (CABG, codes FNA–FNE). From the Cause of Death Register [23] we included data on date of death and underlying cause of death according to ICD-10 until 31 December 2012. Data from the Prescribed Drug Register containing data on dispensed drugs from 2005–2012 was also added in the analyses [24]. Data on educational level (≤9 years, 10–12 years, and ≥12 years of school), income calendar years 2005 and 2009, and country of birth (Sweden, Finland, Nordic countries except Sweden and Finland, European Union except the Nordic countries, Europe except European Union and the Nordic countries, and outside of Europe) was obtained from Statistics Sweden.

### Study participants

**Fig 1:**
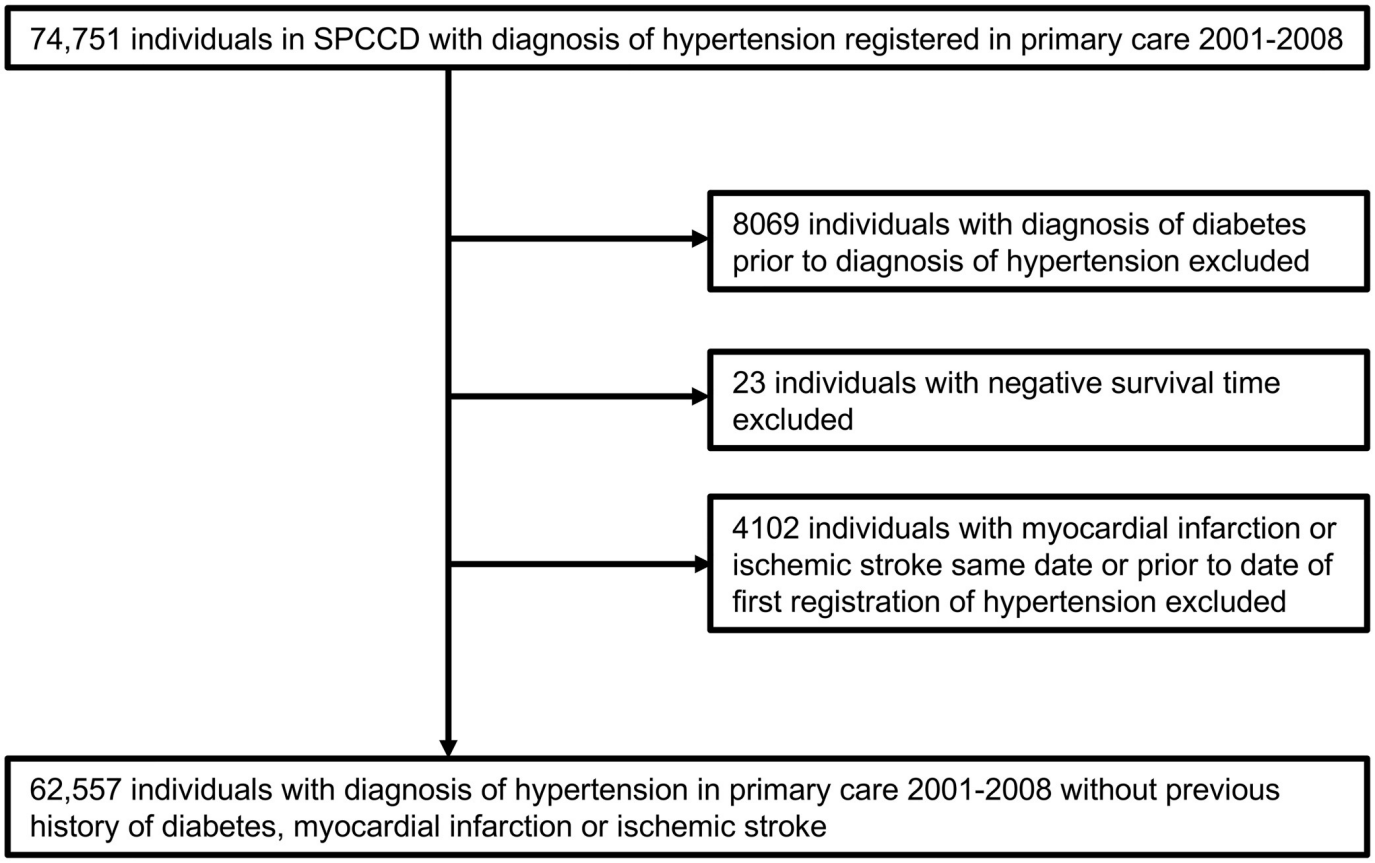
Flowchart of individuals included in the study. Negative survival times could be due to reused or invalid personal identification numbers.

In this cohort study we included all 62,557 individuals in the SPCCD without a recorded diagnosis of myocardial infarction or ischemic stroke in the National Patient Register or diabetes prior to the first diagnosis of hypertension (Fig 1). Diabetes was defined as either a recorded diagnosis from primary care or the National Patient Register, or prescription of antidiabetic medication from primary care 2001–2008. Individuals were included in the study at the first date of registration of a diagnosis of hypertension 2001–2008.

### Outcome assessment and follow up

The study had three outcome variables; all-cause mortality retrieved from the Cause of Death Register, the incidence of myocardial infarction, or ischemic stroke retrieved from the National Patient Register. Individuals were followed-up from study inclusion until the first of the following events: study outcome, death (for study outcome myocardial infarction or ischemic stroke), or end of study 31 December 2012.

### Statistics

Continuous variables were described as mean values ± standard deviation (SD). Categorical variables were described as frequencies and percentages. Missing data were handled by multiple imputation with chained equations, creating 70 imputed datasets [25]. Skewed variables were logarithmically transformed before inclusion in the imputation model and were exponentiated back to their original scale for analysis. The imputation model was stratified for sex and diabetes status during follow up and included: age and calendar year at first diagnosis of hypertension, smoking, BMI, creatinine, SBP, DBP, cholesterol, LDL, HDL, triglycerides, educational level (highest recorded level in either 2005 or 2010), income grouped by quintiles (derived from mean income 2005 and 2009), country of birth, previous PCI and CABG, and comorbidities at baseline recorded in primary care or the National Patient Register (IHD, AF, HF, CVD, TIA, kidney failure and cancer), as well as the event status and the Nelson-Aalen cumulative hazard for each study outcome analyzed.

Number of outcome events and unadjusted incidence rates per 1000 person-years with 95% confidence intervals (CI) for each outcome were calculated stratified for diabetes status, educational level and income grouped by quintiles. The diabetes status was time-updated, i.e. an individual’s follow-up was handled as non-diabetes time until the first date diabetes was recorded and thereafter as diabetes time. Number of outcome events were calculated using complete cases. Outcome incidence rates were calculated by Poisson regression using imputed data.

Cox regression proportional hazards models were used to calculate hazard ratios (HR) to investigate the association between time to study outcomes and educational level, income grouped by quintiles, and time-updated diabetes status as predictors. The analyses were adjusted for multiple covariates using restricted cubic splines with 4 to 7 knots for continuous variables, with the exception of calendar year of study entry which was modelled linear. The number of knots were chosen based on model fit according to the Akaike information criterion. Analyses were performed with age as timescale. In a first model, hazard ratios were adjusted for attained age, sex and calendar year of study entry. In a second model, analyses were additionally adjusted for educational level and income grouped by quintiles. In a third model, analyses were additionally adjusted for country of birth and preexisting conditions at baseline (AF, IHD, HF, CVD, TIA, kidney failure, cancer, PCI and CABG). In the fourth and fully adjusted final model, hazard ratios were additionally adjusted for SBP, DBP, creatinine, smoking, BMI, cholesterol, LDL, HDL and triglycerides. Interaction analyses were performed between diabetes status and sex to check if the outcome HRs of adding diabetes to hypertension were modified by sex. We found no violation of the proportional hazards assumption when evaluating Schoenfeld residuals graphically.

Initial data management was performed using SAS version 9.4 (SAS Institute, Cary, NC, US). The statistical analyses were done using Stata version 15.1 (StataCorp., College station, TX, US). Forest plots were created using R software version 3.5.1 with the package “forestplot”. A two-sided probability value (P) of less than 0.05 was considered statistically significant.

### Ethics

The regional ethical review board in Gothenburg approved the study (references 568–08 and T457-15). The need for individual consent was waived in this retrospective register-based study.

## Results

### Clinical characteristics

The study population is presented in Table 1. Of the 62,557 individuals included in the study, 42% were male and 16% were diagnosed with diabetes during follow-up. The mean age at study entry was 65.0 ± 12.6 years and was lower for individuals with higher educational level or higher income, and gradually increased with lower educational level and income categories. Individuals with diabetes during follow-up were more often men compared to individuals with no diabetes, had higher BMI, lower educational level, lower income and were less frequently born in Sweden. Individuals with >12 years of school compared to ≤9 years of school were younger, more often men, smoked less, had higher income, were more frequently born in a non-European country, and had less cardiovascular comorbidity. Compared to the group with the lowest income, individuals in the highest income group were more often male, were younger, had higher educational level, were less frequently born outside of Sweden, and had less cardiovascular comorbidity. Stroke risks based on the CHA_2_DS_2_-VASc score [26] for individuals with and without diabetes, and study outcome of ischemic stroke are presented in S1 Appendix.

**Table 1:** Characteristics of the study cohort.

	Diabetes status during follow-up		Educational level			Income grouped by quintiles	
	No diabetes	Diabetes	≤9 years	10–12 years	>12 years	Lowest fifth	Highest fifth
Number of individuals	52,576	9981	21,511	22,867	10,087	12,022	12,389
Age at study entry, years	65.0 ± 12.8	65.4 ± 11.4	67.1 ± 11.2	61.0 ± 11.7	59.2 ± 10.9	70.4 ± 12.6	56.5 ± 9.5
Male sex, number	21,535 (41.0)	4862 (48.7)	8892 (41.3)	9825 (43.0)	4492 (44.5)	2659 (22.1)	8062 (65.1)
Systolic blood pressure, mmHg	158.3 ± 21.9	158.8 ± 21.6	160.1 ± 21.9	157.0 ± 21.1	154.9 ± 21.2	161.3 ± 22.7	154.1 ± 20.7
Diastolic blood pressure, mmHg	88.2 ± 11.8	87.1 ± 11.9	87.5 ± 11.7	89.2 ± 11.8	89.4 ± 11.6	86.4 ± 11.9	90.6 ± 11.5
Smoking, yes	2491 (17.1)	888 (16.9)	1296 (16.8)	1475 (19.6)	337 (12.0)	518 (14.0)	588 (16.2)
Weight, kg	78.9 ± 16.4	84.5 ± 17.8	79.9 ± 16.6	82.3 ± 17.2	81.4 ± 16.5	75.4 ± 16.5	87.1 ± 16.8
Length, cm	168.4 ± 9.7	168.3 ± 10.0	167.4 ± 9.8	169.1 ± 9.6	170.8 ± 9.4	162.7 ± 8.7	174.6 ± 9.0
BMI, kg/m^2^	28.2 ± 4.9	30.2 ± 5.4	28.9 ± 5.1	29.0 ± 5.2	28.2 ± 4.9	28.9 ± 5.6	28.8 ± 4.8
Creatinine, mmol/L	83.2 ± 24.2	86.4 ± 24.6	83.3 ± 22.7	80.8 ± 18.7	80.8 ± 21.1	83.7 ± 27.2	82.6 ± 20.2
Cholesterol, mmol/L	5.53 ± 1.03	5.07 ± 1.07	5.43 ± 1.06	5.46 ± 1.04	5.47 ± 1.01	5.51 ± 1.09	5.42 ± 1.00
LDL, mmol/L	3.31 ± 0.90	2.93 ± 0.91	3.22 ± 0.93	3.25 ± 0.90	3.26 ± 0.89	3.26 ± 0.94	3.24 ± 0.88
HDL, mmol/L	1.49 ± 0.42	1.30 ± 0.38	1.44 ± 0.41	1.46 ± 0.43	1.49 ± 0.43	1.48 ± 0.43	1.41 ± 0.40
Triglycerides, mmol/L	1.47 ± 0.77	1.85 ± 1.12	1.57 ± 0.86	1.54 ± 0.88	1.45 ± 0.81	1.61 ± 0.90	1.51 ± 0.87
Educational level							
≤9 years	17,598 (38.2)	3913 (46.6)	NA	NA	NA	5522 (63.7)	2207 (18.0)
10–12 years	19,532 (42.4)	3335 (39.7)	NA	NA	NA	2464 (28.4)	5247 (42.8)
>12 years	8931 (19.4)	1156 (13.7)	NA	NA	NA	678 (7.8)	4804 (39.2)
Income grouped by quintiles							
5 (Highest fifth)	10,836 (21.3)	1553 (16.2)	2207 (10.2)	5247 (22.9)	4804 (47.6)	NA	NA
4	10,363 (20.3)	1813 (18.9)	3432 (16.0)	5900 (25.8)	2495 (24.7)	NA	NA
3	9981 (19.6)	1957 (20.4)	4851 (22.5)	4930 (21.6)	1413 (14.0)	NA	NA
2	9931 (19.5)	2068 (21.5)	5499 (25.6)	4326 (18.9)	697 (6.9)	NA	NA
1 (Lowest fifth)	9813 (19.3)	2209 (23.0)	5522 (25.7)	2464 (10.8)	678 (6.7)	NA	NA
Country of birth							
Sweden	43,034 (81.9)	7708 (77.2)	17,460 (81.2)	18,775 (82.1)	8092 (80.2)	8598 (71.5)	10,765 (86.9)
Finland	3547 (6.7)	747 (7.4)	1752 (8.1)	1706 (7.5)	509 (5.0)	596 (5.0)	757 (6.1)
Other Nordic countries	634 (1.2)	123 (1.2)	202 (0.9)	251 (1.1)	86 (0.8)	203 (1.7)	96 (0.8)
High income Europe	1791 (3.4)	385 (3.9)	511 (2.4)	910 (4.0)	459 (4.6)	454 (3.8)	344 (2.8)
Low income Europe	1346 (2.6)	366 (3.7)	720 (3.4)	489 (2.1)	199 (2.0)	699 (5.8)	142 (1.1)
Non-European	2224 (4.2)	652 (6.6)	866 (4.0)	736 (3.2)	742 (7.4)	1472 (12.2)	285 (2.3)
Comorbidity at baseline							
Ischemic heart disease	3487 (6.6)	624 (6.2)	1620 (7.5)	1227 (5.4)	425 (4.2)	908 (7.5)	484 (3.9)
Atrial fibrillation/flutter	2093 (4.0)	350 (3.5)	842 (3.9)	673 (2.9)	274 (2.7)	511 (4.2)	274 (2.2)
Heart failure	1299 (2.5)	258 (2.6)	503 (2.3)	319 (1.4)	120 (1.2)	461 (3.8)	88 (0.7)
Cerebrovascular disease	1051 (2.0)	155 (1.6)	389 (1.8)	383 (1.7)	154 (1.5)	267 (2.2)	168 (1.4)
Transient ischemic attack	981 (1.9)	119 (1.2)	393 (1.8)	341 (1.5)	158 (1.6)	216 (1.8)	161 (1.3)
Kidney failure	119 (0.2)	12 (0.1)	35 (0.2)	37 (0.2)	15 (0.1)	33 (0.3)	15 (0.1)
PCI	284 (0.5)	55 (0.6)	133 (0.6)	133 (0.6)	49 (0.5)	57 (0.5)	73 (0.6)
CABG	335 (0.6)	75 (0.8)	184 (0.9)	127 (0.6)	44 (0.4)	57 (0.5)	53 (0.4)
Any cardiovascular comorbidity	7184 (13.7)	1211 (12.1)	3076 (14.3)	2477 (10.8)	947 (9.4)	1888 (15.7)	1018 (8.2)
Cancer	3259 (6.2)	441 (4.4)	1159 (5.4)	1255 (5.5)	550 (5.4)	633 (5.3)	568 (4.6)

Abbreviations: BMI, body mass index; LDL, low density lipoprotein; HDL, high density lipoprotein; PCI, percutaneous coronary intervention; CABG, coronary artery bypass grafting; NA, not applicable. Values are expressed as mean ± standard deviation or frequencies (%) if not otherwise specified. High income Europe: European Union except the Nordic countries. Low income Europe: Europe except European Union and the Nordic countries. Systolic and diastolic blood pressure and creatinine are the first recorded values after the first date of diagnosis of hypertension. Smoking, weight, length, BMI, cholesterol, LDL, HDL and triglycerides are the last recorded values. Complete data were available for all individuals regarding age, sex, diabetes status during follow-up, country of birth and comorbidities. Data on systolic and diastolic blood pressure was available for 60,070 (96.0%) individuals; smoking 19,820 (31.7%); weight 26,030 (41.6%); length 19,930 (31.9%); BMI 20,457 (32.7%); creatinine 54,330 (86.8%); cholesterol 50,315 (80.4%); LDL 34,613 (55.3%); HDL 37,290 (59.6%); triglycerides 38,350 (61.3%); educational level 54,465 (87.1%) and income 60,524 (96.8%).

### Unadjusted study outcomes

Number of outcomes and unadjusted outcome incidence rates for all-cause-mortality, myocardial infarction and ischemic stroke stratified for time-updated diabetes status, educational level and income are presented in Table 2. During a median follow up of 8.2 years, 13,231 deaths occurred in 497,493 person-years. During a median follow up of 7.9 years, 4321 myocardial infarctions occurred in 483,438 person-years, and 4433 ischemic strokes in 482,576 person-years. The unadjusted event rates were higher for all study outcomes when hypertension was combined with diabetes, as compared to hypertension without diabetes. Also, all unadjusted event rates increased gradually with declining educational level or declining income, both with and without diabetes.

**Table 2:** Unadjusted study outcome incidence rates per 1000 person-years of follow up.

	All-cause mortality			Myocardial infarction			Ischemic stroke		
	Events	Rate	95% CI	Events	Rate	95% CI	Events	Rate	95% CI
Diabetes status									
No diabetes	10,541	24.4	23.9–24.9	3503	8.3	8.0–8.6	3682	8.8	8.5–9.0
Diabetes	2690	41.0	39.5–42.6	818	13.2	12.3–14.1	751	12.1	11.2–12.9
Educational level									
No diabetes									
>12 years	517	11.6	10.7–12.6	346	5.3	4.8–5.9	305	4.9	4.4–5.4
10–12 years	1659	18.9	18.1–19.7	962	7.1	6.7–7.6	999	7.5	7.1–7.9
≤9 years	2661	35.6	34.6–36.6	1219	10.8	10.3–11.4	1332	11.8	11.3–12.4
Diabetes									
>12 years	112	22.9	19.3–27.2	53	8.4	6.5–10.9	55	8.9	6.8–11.5
10–12 years	465	33.1	30.5–36.1	179	10.4	9.0–11.9	187	10.7	9.4–12.3
≤9 years	790	51.5	48.8–54.3	330	16.6	15.1–18.2	265	13.8	12.5–15.3
Income grouped by quintiles									
No diabetes									
5 (Highest fifth)	498	6.7	6.1–7.3	387	4.5	4.1–5.0	328	3.8	3.4–4.2
4	968	12.7	12.0–13.5	517	6.1	5.6–6.7	511	6.0	5.5–6.6
3	1722	23.0	22.0–24.1	673	8.4	7.8–9.1	731	9.1	8.5–9.8
2	2500	33.4	32.1–34.7	842	10.7	10.0–11.4	924	11.8	11.0–12.5
1 (Lowest fifth)	3276	49.5	47.9–51.1	857	12.5	11.7–13.3	966	14.0	13.2–14.9
Diabetes									
5 (Highest fifth)	141	15.6	13.2–18.3	71	7.4	5.8–9.3	52	5.3	4.0–7.0
4	267	24.8	22.0–27.9	114	10.2	8.5–12.2	112	9.9	8.2–11.9
3	459	37.8	34.5–41.4	157	12.8	10.9–14.9	140	11.3	9.6–13.3
2	632	50.2	46.6–54.2	194	15.4	13.4–17.7	174	13.8	11.9–16.0
1 (Lowest fifth)	826	66.5	62.3–70.9	227	18.1	16.0–20.6	222	17.8	15.7–20.2

Abbreviation: CI, confidence interval. Complete case analyses were used to calculate number of events and imputed data were used to calculate incidence rates.

### Adjusted study outcomes

**Fig 2:**
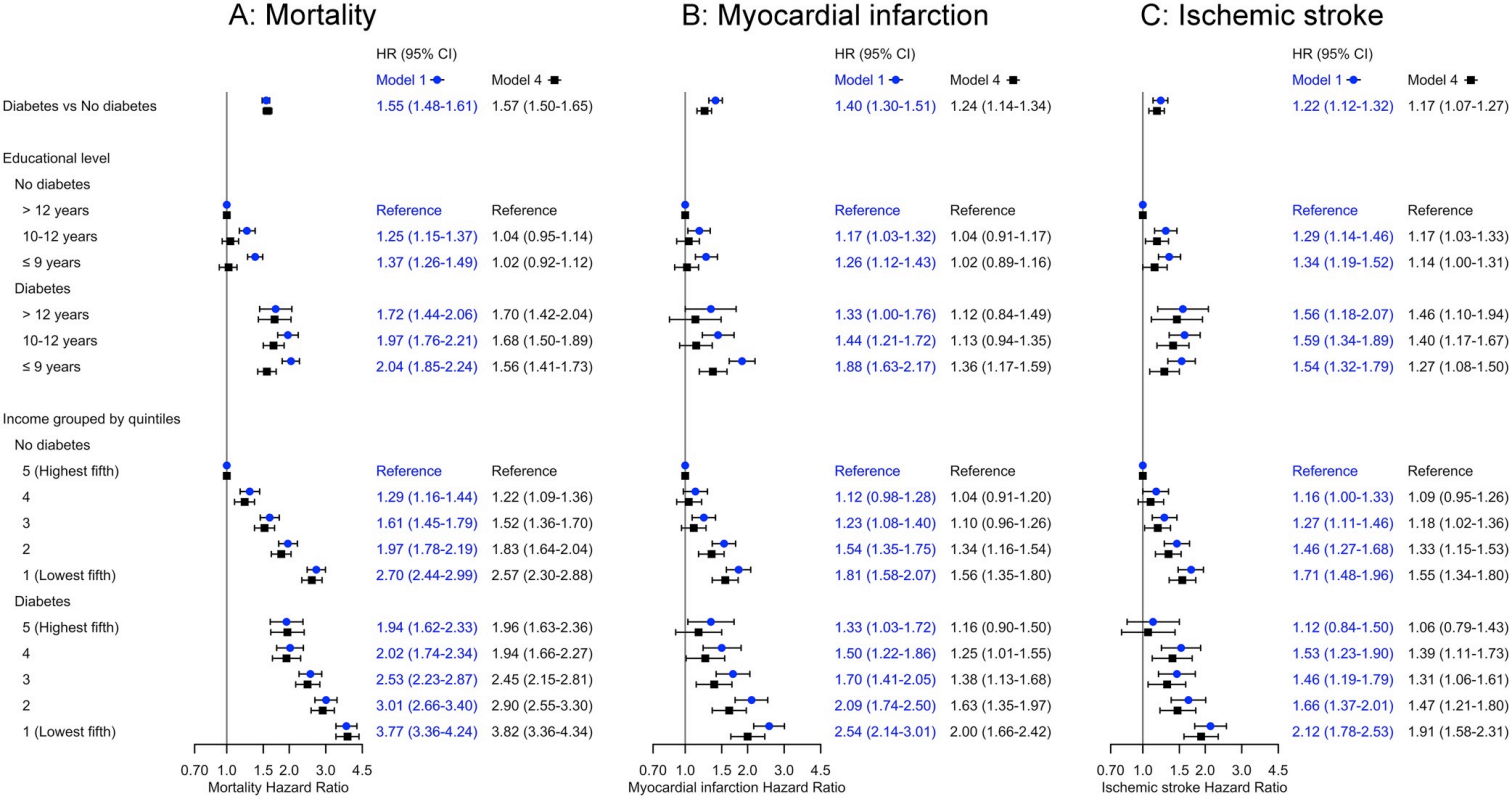
Hazard ratios of study outcomes calculated by Cox regression models. Model 1 was adjusted for sex, attained age and calendar year of first registration of hypertension. Model 4 was additionally adjusted for educational level, income grouped by quintiles, country of birth, preexisting conditions at baseline (atrial fibrillation, ischemic heart disease, heart failure, cerebrovascular disease, transient ischemic cerebral attack, kidney failure, cancer, percutaneous coronary intervention and coronary artery bypass grafting), systolic blood pressure, diastolic blood pressure, creatinine, smoking, body mass index, cholesterol, low density lipoprotein, high density lipoprotein and triglycerides.

Adjusted hazard ratios of the study outcomes mortality, myocardial infarction and ischemic stroke from all 4 Cox regression models are presented in S1–S3 Tables. In the forest-plot in Fig 2, we present the adjusted hazard ratios from the minimally adjusted model 1 and the fully adjusted model 4.

#### All-cause mortality

Adding diabetes to hypertension was associated with increased mortality risk in all models. In model 4, the HR was 1.57 (95% CI 1.50–1.65) and an interaction analysis showed no significant difference in mortality risk between women and men (p for interaction = 0.38). Using the combination of >12 years of school and no diabetes as reference category, the mortality risk in model 1 increased gradually with lower educational level both with and without diabetes with a maximum HR in the lowest education group of 2.04 (95% CI 1.85–2.24) with diabetes and 1.37 (95% CI 1.26–1.49) without diabetes. The association between lower educational level and increased mortality risk could not be shown in model 2 when the model was additionally adjusted for income. Regarding income, using the combination of the highest income group and no diabetes as reference category, the mortality risk increased gradually with declining income level, both with and without diabetes, in all models. In model 4 the largest mortality risk was shown in the lowest income group; HR 3.82 (95% CI 3.36–4.34) with diabetes and 2.57 (95% CI 2.30–2.88) without diabetes.

#### Myocardial infarction

Adding diabetes to hypertension was associated with increased risk of myocardial infarction in all models. In model 4, the HR was 1.24 (95% CI 1.14–1.34) and an interaction analysis showed no significant difference in risk of myocardial infarction between women and men (p for interaction = 0.15). Using the combination of >12 years of school and no diabetes as reference category, the risk of myocardial infarction in model 1 increased gradually with lower educational level, both with and without diabetes with a maximum HR in the lowest education group of 1.88 (95% CI 1.63–2.17) with diabetes and 1.26 (95% CI 1.12–1.43) without diabetes. Model 2–4 showed no, or weaker associations between lower educational level and increased risk of myocardial infarction. Regarding income, using the combination of the highest income group and no diabetes as reference category, the risk of myocardial infarction increased gradually with declining income level both with and without diabetes in all models. In model 4 the largest risk of myocardial infarction was shown in the lowest income group; HR 2.00 (95% CI 1.66–2.42) with diabetes and 1.56 (95% CI 1.35–1.80) without diabetes.

#### Ischemic stroke

Adding diabetes to hypertension was associated with increased risk of ischemic stroke in all models. In model 4, the HR was 1.17 (95% CI 1.07–1.27) and an interaction analysis showed no significant difference in risk of ischemic stroke between women and men (p for interaction = 0.53). Using the combination of >12 years of school and no diabetes as reference category, the risk of ischemic stroke was similarly increased across all educational levels in combination with diabetes with maximum HR 1.46 (95% CI 1.10–1.94) in the >12 years category in model 4. The risk of ischemic stroke was similar in the two lowest educational level categories without diabetes with maximum HR 1.17 (95% CI 1.03–1.33) for 10–12 years of school in model 4. Regarding income, using the combination of the highest income group and no diabetes as reference category, the risk of ischemic stroke increased gradually with declining income level, both with and without diabetes in model 1 and 4. In model 4, the largest risk of ischemic stroke was shown in the lowest income group; HR 1.91 (95% CI 1.58–2.31) with diabetes and 1.55 (95% CI 1.34–1.80) without diabetes.

## Discussion

In this Swedish register-based cohort study of 62,557 individuals with hypertension in primary care, adding diabetes to hypertension was associated with 57% excess risk of mortality, 24% excess risk of myocardial infarction and 17% excess risk of ischemic stroke. The mortality risk was nearly 4-fold and the risk of myocardial infarction and ischemic stroke 2-fold when contrasting the combination of hypertension, diabetes and income in the lowest fifth group versus hypertension and income in the highest fifth group.

### Strengths and limitations of the study

This study has several strengths. First, we studied a large unselected primary care cohort from two geographical areas using routine medical records representing every-day clinical practice, thus limiting the risk of selection bias that would arise in a secondary or tertiary care setting. Second, high quality national registers were used to assess study outcomes limiting the risk of undetected outcomes. Third, diabetes status was used as a time-updated variable reducing the risk of immortal time bias [27]. Fourth, we have been able to adjust study outcomes for several potential confounders, including individual clinical data and important comorbidities, for example heart failure [7]. Some weaknesses also need to be addressed. First, since this is an observational study, we cannot show causality between predictors and outcomes. Second, as the SPCCD includes all individuals with diagnosed hypertension attending primary care, individuals with hypertension but without ICD-10 diagnosis of hypertension in the medical records were not included in the study. Also, the diagnosis of hypertension was determined by the individual physician. However, the validity of the diagnosis of hypertension in the SPCCD has previously been shown to be high [28]. Third, individuals with diabetes in the study comprise a mix of type 1 and type 2 diabetes, which could not be methodologically distinguished due to potential misclassifications of ICD-diagnoses, i.e. an individual with insulin treated type 2 diabetes could be misclassified as type 1 diabetes. However, type 2 diabetes represents 85–90% of diabetes in Sweden [29] and the proportion is estimated to be even higher in the present study as only individuals ≥30 years old with a diagnosis of hypertension from primary care were included in the study, and the vast majority of individuals with type 1 diabetes are managed in hospital based secondary care clinics. Consequently, the results in the current study are in practice reflecting type 2 diabetes. Fourth, as often encountered in register-based studies from routine care, there is a varying degree of missing data. However, multiple imputations were used to reduce the impact of missing data. Fifth, residual confounders cannot be ruled out, e.g. other relevant comorbidities, medications and socioeconomic factors such as marital status, occupational status and area deprivation. Sixth, although the study findings are likely to be applicable on a national basis, they could possibly differ in another study setting with respect to mix of ethnicities, societal socio-economy and health care systems.

### Comparison with previous studies

The excess risk of mortality, myocardial infarction and ischemic stroke associated with adding diabetes to hypertension in our study is in line with findings from other studies. In a Swedish population based study with up to 28-years follow-up of hypertensive middle-aged men, new-onset diabetes was associated with increased adjusted risk of mortality (HR 1.42), myocardial infarction (HR 1.66) and stroke (HR 1.67) [30]. A Scottish study with 40-years of follow-up from a secondary and tertiary level hypertension clinic showed increased adjusted risk of mortality among individuals with coexisting prevalent diabetes (HR 1.84) and among individuals with early new-onset diabetes (HR 1.39), defined as diabetes occurring within 10-years after study inclusion. In contrast, no excess mortality was seen in individuals with late new-onset diabetes [31]. Partially conflicting results were shown in a post-hoc analysis from the Valsartan Antihypertensive Long-term Use Evaluation randomized clinical trial including more than 15,000 individuals with hypertension and high cardiovascular risk followed up for a median of 4.2 years [32]. In this study, diabetes at baseline was associated with significantly increased risk of mortality (HR 1.41), myocardial infarction (HR 1.73) and stroke (HR 1.27), whereas new-onset diabetes was associated with significantly lower mortality risk (HR 0.61) and non-significantly increased risk of myocardial infarction and stroke. These unexpected results could, as acknowledged by the authors, partially be explained by immortal-time bias.

We studied the effect of educational level and income on the risk for mortality, myocardial infarction and ischemic stroke. Low income was consistently and strongly associated with increased risk of all outcomes in all models, also after adjusting for educational level. Low educational level was in most cases only associated with increased risk when no adjustment for income was applied. However, an effect of education would likely be partially mediated by income, and hence it is difficult to separate the effects of education and income on the outcomes [33]. Our results regarding mortality in individuals with hypertension and diabetes are in line with a previous nationwide Swedish study of individuals with type 2 diabetes [18]. In that study, high versus low educational level was associated with decreased mortality risk, and the mortality risk also increased gradually with declining income. Comparable results were shown in a Danish nationwide observational register-based study including individuals with and without diabetes [34]. Additional aspects of the association between socio-economic position and mortality were explored in a Finnish observational study of individuals with diabetes [17]. In that study low occupational position, unemployment and living alone were found to be associated with worse outcome in addition to the factors of low educational level and income.

In our study, the magnitude of the increased risk of mortality and cardiovascular events associated with low income was larger, and regarding low educational level similar, as when adding diabetes to hypertension. Worst outcome was seen for individuals with hypertension in combination with low income and diabetes. These findings suggest that it might be of importance to give extra focus on individuals with hypertension and low socioeconomic status in clinical care. This may include aggressive risk factor management especially among individuals with diabetes, where significant benefits regarding clinical outcomes in relation to multifactorial risk factor treatment have been seen in observational [13] as well as in clinical randomized studies [35]. Of note, the Swedish subsidized universal healthcare system is quite equitable compared to many other countries, with low individual costs for clinic visits and drugs [36]. Therefore, a health care access bias is less likely to be a sufficient explanation for the association between low socioeconomic status and adverse outcomes in the current study. It has been shown that psychosocial factors, including financial stress, are associated with increased risk of myocardial infarction [37], and that low income status is associated with nonadherence to antihypertensive treatment [38]. Other possible explanations put forward in previous studies include that absence of socioeconomic privilege is associated with unhealthy behaviors such as sedentary lifestyle, smoking, alcohol use and poor nutrition as well as inequality, stress, lack of social support and lack of knowledge and access to information about health risks [39]. In addition, neighborhood deprivation has been associated with increased risk of mortality [40] and stroke [41] even after adjustment for multiple other socioeconomic factors.

### Conclusion

Concomitant diabetes in individuals with hypertension attending primary care was associated with an excess risk of mortality, myocardial infarction, and ischemic stroke. Low income in addition to diabetes was associated with up to 4-fold risk of mortality and 2-fold risk of myocardial infarction and ischemic stroke, and we therefore propose that extra care should be taken by clinicians and policy makers to ensure sufficient risk factor control among those patients. This includes promoting lifestyle changes and better control of blood pressure, lipids, and glucose.

## Supporting information

S1 Appendix CHA_2_DS_2_-VASc score calculation for individuals with and without diabetes, and study outcome ischemic stroke. (DOCX)

S1 Table Association between risk of mortality and diabetes status, educational level and income. (DOCX)

S2 Table Association between risk of myocardial infarction and diabetes status, educational level and income. (DOCX)

S3 Table Association between risk of ischemic stroke and diabetes status, educational level and income. (DOCX)
