# Supplementary material for: The impact of diabetes, education and income on mortality and cardiovascular events in hypertensive patients: A cohort study from the Swedish Primary Care Cardiovascular Database (SPCCD)
Source: PLoS One. 2020 Aug 3;15(8):e0237107. doi: 10.1371/journal.pone.0237107 (PMC7398497; doi:10.1371/journal.pone.0237107)
Supplement: S1 Appendix — (DOCX) [file pone.0237107.s001.docx]

**S1 Appendix. CHA_2_DS_2_-VASc score calculation for individuals with and without diabetes, and study outcome ischemic stroke.**

For individuals with an event of ischemic stroke during follow-up, we used available study data to calculate approximate CHA_2_DS_2_-VASc scores at the time point of the first ischemic stroke among individuals with and without diabetes. However, the scores are presumably underestimated to some extent due to missing data regarding diagnoses of *thromboembolism* in the “**S**troke” component*,* and *peripheral artery disease* or *aortic plaque* in the “**V**ascular disease” component.

**Table 1. ICD-10 diagnoses used to calculate CHA_2_DS_2_-VASc scores for individuals with and without diabetes, and study outcome of ischemic stroke.**

|  | **Points** | **ICD-10 diagnosis** | **Number of individuals** | |
| --- | --- | --- | --- | --- |
|  |  |  | **With diabetes (n=751)** | **Without diabetes (n=3682)** |
| **C**ongestive heart failure | 1 | I50 | 166 | 621 |
| **H**ypertension | 1 | I10, I13P, I15 | 751 | 3682 |
| **A**ge 75 years or older | 2 |  | 454 | 2460 |
| **D**iabetes | 1 | E10-11, E14 | 751 | 0 |
| **S**troke, transient ischemic attack, or thromboembolism | 2 | I63, G45 | 59 | 340 |
| **V**ascular disease | 1 | I21 | 66 | 196 |
| **A**ge 65-74 years | 1 |  | 192 | 762 |
| **S**ex **c**ategory (female) | 1 |  | 391 | 2106 |

**Table 2. CHA_2_DS_2_-VASc scores for individuals with and without diabetes, and study outcome of ischemic stroke.**

|  | **CHA_2_DS_2-_VASc score** |  |  |
| --- | --- | --- | --- |
|  | Median (interquartile range) | Minimum | Maximum |
| With diabetes (n=751) | 4 (4–5) | 2 | 9 |
| Female (n=391) | 5 (4–6) | 3 | 9 |
| Male (n=360) | 4 (3–4) | 2 | 8 |
| Without diabetes (n=3682) | 4 (3–4) | 1 | 8 |
| Female (n=2106) | 4 (4–4) | 2 | 8 |
| Male (n=1576) | 3 (2–3) | 1 | 7 |
