# Supplementary material for: The impact of diabetes, education and income on mortality and cardiovascular events in hypertensive patients: A cohort study from the Swedish Primary Care Cardiovascular Database (SPCCD)
Source: PLoS One. 2020 Aug 3;15(8):e0237107. doi: 10.1371/journal.pone.0237107 (PMC7398497; doi:10.1371/journal.pone.0237107)
Supplement: S1 Table — (DOCX) [file pone.0237107.s002.docx]

**S1 Table.** Association between risk of mortality and diabetes status, educational level and income.

|  | **Model 1** | | | **Model 2** | | | **Model 3** | | | **Model 4** |  |  |
| --- | --- | --- | --- | --- | --- | --- | --- | --- | --- | --- | --- | --- |
|  | **HR** | **95% CI** | **p-value** | **HR** | **95% CI** | **p-value** | **HR** | **95% CI** | **p-value** | **HR** | **95% CI** | **p-value** |
| **Diabetes vs no diabetes** | 1.55 | 1.48–1.61 | <0.001 | 1.51 | 1.44–1.57 | <0.001 | 1.56 | 1.49–1.62 | <0.001 | 1.57 | 1.50–1.65 | <0.001 |
| **Education** |  |  |  |  |  |  |  |  |  |  |  |  |
| No diabetes |  |  |  |  |  |  |  |  |  |  |  |  |
| >12 years | reference |  |  | reference |  |  | reference |  |  | reference |  |  |
| 10–12 years | 1.25 | 1.15–1.37 | <0.001 | 1.09 | 1.00–1.19 | 0.061 | 1.06 | 0.97–1.16 | 0.17 | 1.04 | 0.95–1.14 | 0.39 |
| ≤9 years | 1.37 | 1.26–1.49 | <0.001 | 1.06 | 0.96–1.16 | 0.23 | 1.04 | 0.94–1.14 | 0.46 | 1.02 | 0.92–1.12 | 0.76 |
| Diabetes |  |  |  |  |  |  |  |  |  |  |  |  |
| >12 years | 1.72 | 1.44–2.06 | <0.001 | 1.62 | 1.36–1.94 | <0.001 | 1.70 | 1.42–2.04 | <0.001 | 1.70 | 1.42–2.04 | <0.001 |
| 10–12 years | 1.97 | 1.76–2.21 | <0.001 | 1.69 | 1.51–1.89 | <0.001 | 1.71 | 1.52–1.91 | <0.001 | 1.68 | 1.50–1.89 | <0.001 |
| ≤9 years | 2.04 | 1.85–2.24 | <0.001 | 1.56 | 1.41–1.72 | <0.001 | 1.57 | 1.42–1.74 | <0.001 | 1.56 | 1.41–1.73 | <0.001 |
| **Income grouped by quintiles** |  |  |  |  |  |  |  |  |  |  |  |  |
| No diabetes |  |  |  |  |  |  |  |  |  |  |  |  |
| 5 (Highest fifth) | reference |  |  | reference |  |  | reference |  |  | reference |  |  |
| 4 | 1.29 | 1.16–1.44 | <0.001 | 1.28 | 1.15–1.43 | <0.001 | 1.28 | 1.15–1.43 | <0.001 | 1.22 | 1.09–1.36 | 0.001 |
| 3 | 1.61 | 1.45–1.79 | <0.001 | 1.60 | 1.44–1.78 | <0.001 | 1.61 | 1.45–1.80 | <0.001 | 1.52 | 1.36–1.70 | <0.001 |
| 2 | 1.97 | 1.78–2.19 | <0.001 | 1.95 | 1.75–2.18 | <0.001 | 1.98 | 1.77–2.20 | <0.001 | 1.83 | 1.64–2.04 | <0.001 |
| 1 (Lowest fifth) | 2.70 | 2.44–2.99 | <0.001 | 2.69 | 2.41–2.99 | <0.001 | 2.79 | 2.50–3.11 | <0.001 | 2.57 | 2.30–2.88 | <0.001 |
| Diabetes |  |  |  |  |  |  |  |  |  |  |  |  |
| 5 (Highest fifth) | 1.94 | 1.62–2.33 | <0.001 | 1.93 | 1.61–2.32 | <0.001 | 1.97 | 1.64–2.37 | <0.001 | 1.96 | 1.63–2.36 | <0.001 |
| 4 | 2.02 | 1.74–2.34 | <0.001 | 2.00 | 1.72–2.32 | <0.001 | 2.04 | 1.75–2.37 | <0.001 | 1.94 | 1.66–2.27 | <0.001 |
| 3 | 2.53 | 2.23–2.87 | <0.001 | 2.51 | 2.20–2.86 | <0.001 | 2.59 | 2.27–2.95 | <0.001 | 2.45 | 2.15–2.81 | <0.001 |
| 2 | 3.01 | 2.66–3.40 | <0.001 | 2.99 | 2.63–3.39 | <0.001 | 3.09 | 2.72–3.51 | <0.001 | 2.90 | 2.55–3.30 | <0.001 |
| 1 (Lowest fifth) | 3.77 | 3.36–4.24 | <0.001 | 3.76 | 3.33–4.24 | <0.001 | 4.08 | 3.61–4.62 | <0.001 | 3.82 | 3.36–4.34 | <0.001 |

HR: hazard ratio, 95% CI: 95% confidence interval
Model 1 adjusted for sex, attained age, calendar year of study entry
Model 2 adjusted for same as model 1 + educational level and income
Model 3 adjusted same as model 2 + country of birth and comorbidities (ischemic heart disease, atrial fibrillation/flutter, heart failure, cerebrovascular disease, transient cerebral ischemic attack, kidney failure, percutaneous coronary intervention, coronary artery bypass grafting, cancer)
Model 4 adjusted for same as model 3 + smoking, body mass index, creatinine, systolic blood pressure, diastolic blood pressure, cholesterol, low density lipoprotein, high density lipoprotein, triglycerides
