# Supplementary material for: The impact of diabetes, education and income on mortality and cardiovascular events in hypertensive patients: A cohort study from the Swedish Primary Care Cardiovascular Database (SPCCD)
Source: PLoS One. 2020 Aug 3;15(8):e0237107. doi: 10.1371/journal.pone.0237107 (PMC7398497; doi:10.1371/journal.pone.0237107)
Supplement: S2 Table — (DOCX) [file pone.0237107.s003.docx]

**S2 Table.** Association between risk of myocardial infarction and diabetes status, educational level and income.

|  | **Model 1** | | | **Model 2** | | | **Model 3** | | | **Model 4** |  |  |
| --- | --- | --- | --- | --- | --- | --- | --- | --- | --- | --- | --- | --- |
|  | **HR** | **95% CI** | **p-value** | **HR** | **95% CI** | **p-value** | **HR** | **95% CI** | **p-value** | **HR** | **95% CI** | **p-value** |
| **Diabetes vs no diabetes** | 1.40 | 1.30–1.51 | <0.001 | 1.37 | 1.27–1.48 | <0.001 | 1.39 | 1.29–1.50 | <0.001 | 1.24 | 1.14–1.34 | <0.001 |
| **Education** |  |  |  |  |  |  |  |  |  |  |  |  |
| No diabetes |  |  |  |  |  |  |  |  |  |  |  |  |
| >12 years | reference |  |  | reference |  |  | reference |  |  | reference |  |  |
| 10–12 years | 1.17 | 1.03–1.32 | 0.013 | 1.07 | 0.95–1.22 | 0.25 | 1.06 | 0.94–1.20 | 0.34 | 1.04 | 0.91–1.17 | 0.59 |
| ≤9 years | 1.26 | 1.12–1.43 | <0.001 | 1.08 | 0.95–1.23 | 0.26 | 1.06 | 0.94–1.21 | 0.35 | 1.02 | 0.89–1.16 | 0.81 |
| Diabetes |  |  |  |  |  |  |  |  |  |  |  |  |
| >12 years | 1.33 | 1.00–1.76 | 0.050 | 1.27 | 0.96–1.69 | 0.094 | 1.28 | 0.96–1.70 | 0.087 | 1.12 | 0.84–1.49 | 0.44 |
| 10–12 years | 1.44 | 1.21–1.72 | <0.001 | 1.31 | 1.10–1.57 | 0.003 | 1.31 | 1.10–1.57 | 0.003 | 1.13 | 0.94–1.35 | 0.19 |
| ≤9 years | 1.88 | 1.63–2.17 | <0.001 | 1.59 | 1.37–1.84 | <0.001 | 1.60 | 1.37–1.86 | <0.001 | 1.36 | 1.17–1.59 | <0.001 |
| **Income grouped by quintiles** |  |  |  |  |  |  |  |  |  |  |  |  |
| No diabetes |  |  |  |  |  |  |  |  |  |  |  |  |
| 5 (Highest fifth) | reference |  |  | reference |  |  | reference |  |  | reference |  |  |
| 4 | 1.12 | 0.98–1.28 | 0.10 | 1.10 | 0.96–1.26 | 0.15 | 1.09 | 0.95–1.25 | 0.20 | 1.04 | 0.91–1.20 | 0.54 |
| 3 | 1.23 | 1.08–1.40 | 0.002 | 1.19 | 1.04–1.37 | 0.010 | 1.18 | 1.03–1.35 | 0.020 | 1.10 | 0.96–1.26 | 0.16 |
| 2 | 1.54 | 1.35–1.75 | <0.001 | 1.49 | 1.30–1.70 | <0.001 | 1.46 | 1.28–1.68 | <0.001 | 1.34 | 1.16–1.54 | <0.001 |
| 1 (Lowest fifth) | 1.81 | 1.58–2.07 | <0.001 | 1.74 | 1.51–2.00 | <0.001 | 1.74 | 1.51–2.01 | <0.001 | 1.56 | 1.35–1.80 | <0.001 |
| Diabetes |  |  |  |  |  |  |  |  |  |  |  |  |
| 5 (Highest fifth) | 1.33 | 1.03–1.72 | 0.026 | 1.32 | 1.03–1.71 | 0.030 | 1.33 | 1.03–1.71 | 0.028 | 1.16 | 0.90–1.50 | 0.25 |
| 4 | 1.50 | 1.22–1.86 | <0.001 | 1.48 | 1.20–1.83 | <0.001 | 1.47 | 1.19–1.82 | <0.001 | 1.25 | 1.01–1.55 | 0.040 |
| 3 | 1.70 | 1.41–2.05 | <0.001 | 1.65 | 1.36–2.00 | <0.001 | 1.64 | 1.35–1.99 | <0.001 | 1.38 | 1.13–1.68 | 0.002 |
| 2 | 2.09 | 1.74–2.50 | <0.001 | 2.01 | 1.68–2.42 | <0.001 | 2.02 | 1.68–2.44 | <0.001 | 1.63 | 1.35–1.97 | <0.001 |
| 1 (Lowest fifth) | 2.54 | 2.14–3.01 | <0.001 | 2.45 | 2.05–2.92 | <0.001 | 2.50 | 2.09–3.00 | <0.001 | 2.00 | 1.66–2.42 | <0.001 |

HR: hazard ratio, 95% CI: 95% confidence interval
Model 1 adjusted for sex, attained age, calendar year of study entry
Model 2 adjusted for same as model 1 + educational level and income
Model 3 adjusted same as model 2 + country of birth and comorbidities (ischemic heart disease, atrial fibrillation/flutter, heart failure, cerebrovascular disease, transient cerebral ischemic attack, kidney failure, percutaneous coronary intervention, coronary artery bypass grafting, cancer)
Model 4 adjusted for same as model 3 + smoking, body mass index, creatinine, systolic blood pressure, diastolic blood pressure, cholesterol, low density lipoprotein, high density lipoprotein, triglycerides
