# Supplementary material for: The impact of diabetes, education and income on mortality and cardiovascular events in hypertensive patients: A cohort study from the Swedish Primary Care Cardiovascular Database (SPCCD)
Source: PLoS One. 2020 Aug 3;15(8):e0237107. doi: 10.1371/journal.pone.0237107 (PMC7398497; doi:10.1371/journal.pone.0237107)
Supplement: S3 Table — (DOCX) [file pone.0237107.s004.docx]

**S3 Table.** Association between risk of ischemic stroke and diabetes status, educational level and income.

|  | **Model 1** | | | **Model 2** | | | **Model 3** | | | **Model 4** |  |  |
| --- | --- | --- | --- | --- | --- | --- | --- | --- | --- | --- | --- | --- |
|  | **HR** | **95% CI** | **p-value** | **HR** | **95% CI** | **p-value** | **HR** | **95% CI** | **p-value** | **HR** | **95% CI** | **p-value** |
| **Diabetes vs no diabetes** | 1.22 | 1.12–1.32 | <0.001 | 1.19 | 1.10–1.29 | <0.001 | 1.22 | 1.12–1.32 | <0.001 | 1.17 | 1.07–1.27 | <0.001 |
| **Education** |  |  |  |  |  |  |  |  |  |  |  |  |
| No diabetes |  |  |  |  |  |  |  |  |  |  |  |  |
| >12 years | reference |  |  | reference |  |  | reference |  |  | reference |  |  |
| 10–12 years | 1.29 | 1.14–1.46 | <0.001 | 1.19 | 1.05–1.35 | 0.007 | 1.19 | 1.05–1.35 | 0.008 | 1.17 | 1.03–1.33 | 0.015 |
| ≤9 years | 1.34 | 1.19–1.52 | <0.001 | 1.17 | 1.02–1.34 | 0.021 | 1.17 | 1.02–1.34 | 0.022 | 1.14 | 1.00–1.31 | 0.049 |
| Diabetes |  |  |  |  |  |  |  |  |  |  |  |  |
| >12 years | 1.56 | 1.18–2.07 | 0.002 | 1.52 | 1.14–2.01 | 0.004 | 1.54 | 1.16–2.05 | 0.003 | 1.46 | 1.10–1.94 | 0.009 |
| 10–12 years | 1.59 | 1.34–1.89 | <0.001 | 1.46 | 1.23–1.74 | <0.001 | 1.49 | 1.25–1.77 | <0.001 | 1.40 | 1.17–1.67 | <0.001 |
| ≤9 years | 1.54 | 1.32–1.79 | <0.001 | 1.33 | 1.13–1.56 | 0.001 | 1.35 | 1.15–1.59 | <0.001 | 1.27 | 1.08–1.50 | 0.004 |
| **Income grouped by quintiles** |  |  |  |  |  |  |  |  |  |  |  |  |
| No diabetes |  |  |  |  |  |  |  |  |  |  |  |  |
| 5 (Highest fifth) | reference |  |  | reference |  |  | reference |  |  | reference |  |  |
| 4 | 1.16 | 1.00–1.33 | 0.043 | 1.14 | 0.99–1.31 | 0.078 | 1.13 | 0.98–1.30 | 0.096 | 1.09 | 0.95–1.26 | 0.22 |
| 3 | 1.27 | 1.11–1.46 | 0.001 | 1.24 | 1.07–1.42 | 0.003 | 1.23 | 1.07–1.42 | 0.004 | 1.18 | 1.02–1.36 | 0.022 |
| 2 | 1.46 | 1.27–1.68 | <0.001 | 1.41 | 1.22–1.63 | <0.001 | 1.40 | 1.21–1.62 | <0.001 | 1.33 | 1.15–1.53 | <0.001 |
| 1 (Lowest fifth) | 1.71 | 1.48–1.96 | <0.001 | 1.65 | 1.43–1.91 | <0.001 | 1.65 | 1.42–1.91 | <0.001 | 1.55 | 1.34–1.80 | <0.001 |
| Diabetes |  |  |  |  |  |  |  |  |  |  |  |  |
| 5 (Highest fifth) | 1.12 | 0.84–1.50 | 0.45 | 1.11 | 0.83–1.49 | 0.48 | 1.13 | 0.84–1.51 | 0.42 | 1.06 | 0.79–1.43 | 0.68 |
| 4 | 1.53 | 1.23–1.90 | <0.001 | 1.49 | 1.20–1.85 | <0.001 | 1.50 | 1.20–1.86 | <0.001 | 1.39 | 1.11–1.73 | 0.004 |
| 3 | 1.46 | 1.19–1.79 | <0.001 | 1.41 | 1.15–1.74 | 0.001 | 1.43 | 1.16–1.75 | 0.001 | 1.31 | 1.06–1.61 | 0.011 |
| 2 | 1.66 | 1.37–2.01 | <0.001 | 1.61 | 1.32–1.95 | <0.001 | 1.61 | 1.33–1.97 | <0.001 | 1.47 | 1.21–1.80 | <0.001 |
| 1 (Lowest fifth) | 2.12 | 1.78–2.53 | <0.001 | 2.06 | 1.71–2.47 | <0.001 | 2.10 | 1.74–2.54 | <0.001 | 1.91 | 1.58–2.31 | <0.001 |

HR: hazard ratio, 95% CI: 95% confidence interval
Model 1 adjusted for sex, attained age, calendar year of study entry
Model 2 adjusted for same as model 1 + educational level and income
Model 3 adjusted same as model 2 + country of birth and comorbidities (ischemic heart disease, atrial fibrillation/flutter, heart failure, cerebrovascular disease, transient cerebral ischemic attack, kidney failure, percutaneous coronary intervention, coronary artery bypass grafting, cancer)
Model 4 adjusted for same as model 3 + smoking, body mass index, creatinine, systolic blood pressure, diastolic blood pressure, cholesterol, low density lipoprotein, high density lipoprotein, triglycerides
